# Supplementary material for: Modeling glioblastoma heterogeneity as a dynamic network of cell states
Source: Mol Syst Biol. 2021 Sep 16;17(9):e10105. doi: 10.15252/msb.202010105 (PMC8444284; doi:10.15252/msb.202010105)
Supplement: Supplementary file 6 — Source Data for Figure 5 [file MSB-17-e10105-s004.zip › Figure5A_sourcedata/GSEA_3017/nosigdb_stateB.GseaPreranked.1622795021725/FELDMAN_2020_S-G2.html]

Details for gene set FELDMAN\_2020\_S-G2[GSEA]

|  || Dataset | state43017 |
| Phenotype | NoPhenotypeAvailable |
| Upregulated in class | na\_pos |
| GeneSet | FELDMAN\_2020\_S-G2 |
| Enrichment Score (ES) | 0.56223416 |
| Normalized Enrichment Score (NES) | 3.5360239 |
| Nominal p-value | 0.0 |
| FDR q-value | 0.0 |
| FWER p-Value | 0.0 |
Table: GSEA Results Summary

  

Fig 1: Enrichment plot: FELDMAN\_2020\_S-G2      
 Profile of the Running ES Score & Positions of GeneSet Members on the Rank Ordered List

  

| PROBE | GENE SYMBOL | GENE\_TITLE | RANK IN GENE LIST | RANK METRIC SCORE | RUNNING ES | CORE ENRICHMENT || 1 | HIST1H4C |  |  | 0 | 1.373 | 0.0160 | Yes |
| 2 | HIST1H1B |  |  | 1 | 1.272 | 0.0308 | Yes |
| 3 | HIST1H1D |  |  | 2 | 1.180 | 0.0446 | Yes |
| 4 | TOP2A |  |  | 3 | 1.079 | 0.0572 | Yes |
| 5 | HIST1H1C |  |  | 4 | 1.041 | 0.0693 | Yes |
| 6 | HIST1H1E |  |  | 5 | 0.983 | 0.0807 | Yes |
| 7 | HIST1H2AH |  |  | 6 | 0.883 | 0.0910 | Yes |
| 8 | RRM2 |  |  | 7 | 0.875 | 0.1012 | Yes |
| 9 | HIST2H2AC |  |  | 9 | 0.872 | 0.1097 | Yes |
| 10 | UBE2C |  |  | 11 | 0.802 | 0.1173 | Yes |
| 11 | TPX2 |  |  | 12 | 0.801 | 0.1267 | Yes |
| 12 | NUSAP1 |  |  | 13 | 0.801 | 0.1360 | Yes |
| 13 | HMGB2 |  |  | 14 | 0.786 | 0.1452 | Yes |
| 14 | FBXO5 |  |  | 15 | 0.785 | 0.1543 | Yes |
| 15 | NCAPG |  |  | 16 | 0.778 | 0.1634 | Yes |
| 16 | SPC25 |  |  | 17 | 0.763 | 0.1723 | Yes |
| 17 | PRC1 |  |  | 18 | 0.756 | 0.1811 | Yes |
| 18 | CDCA2 |  |  | 19 | 0.751 | 0.1898 | Yes |
| 19 | ESCO2 |  |  | 20 | 0.742 | 0.1985 | Yes |
| 20 | TACC3 |  |  | 21 | 0.741 | 0.2071 | Yes |
| 21 | GTSE1 |  |  | 22 | 0.732 | 0.2157 | Yes |
| 22 | HJURP |  |  | 23 | 0.731 | 0.2242 | Yes |
| 23 | KIF23 |  |  | 24 | 0.721 | 0.2326 | Yes |
| 24 | ASPM |  |  | 26 | 0.695 | 0.2390 | Yes |
| 25 | NDC80 |  |  | 27 | 0.695 | 0.2471 | Yes |
| 26 | ATAD2 |  |  | 28 | 0.685 | 0.2550 | Yes |
| 27 | ARHGAP11A |  |  | 30 | 0.683 | 0.2613 | Yes |
| 28 | KPNA2 |  |  | 33 | 0.676 | 0.2658 | Yes |
| 29 | CENPF |  |  | 34 | 0.670 | 0.2736 | Yes |
| 30 | KIF4A |  |  | 37 | 0.662 | 0.2779 | Yes |
| 31 | ANLN |  |  | 39 | 0.659 | 0.2839 | Yes |
| 32 | RAD51AP1 |  |  | 40 | 0.657 | 0.2915 | Yes |
| 33 | SMC4 |  |  | 41 | 0.655 | 0.2992 | Yes |
| 34 | MELK |  |  | 42 | 0.655 | 0.3068 | Yes |
| 35 | CENPE |  |  | 43 | 0.654 | 0.3144 | Yes |
| 36 | NUF2 |  |  | 44 | 0.651 | 0.3220 | Yes |
| 37 | KIFC1 |  |  | 47 | 0.645 | 0.3261 | Yes |
| 38 | DHFR |  |  | 49 | 0.638 | 0.3319 | Yes |
| 39 | CENPA |  |  | 51 | 0.636 | 0.3376 | Yes |
| 40 | CKAP2 |  |  | 53 | 0.628 | 0.3432 | Yes |
| 41 | CCNA2 |  |  | 54 | 0.626 | 0.3505 | Yes |
| 42 | MYBL2 |  |  | 58 | 0.612 | 0.3525 | Yes |
| 43 | CKAP2L |  |  | 59 | 0.611 | 0.3596 | Yes |
| 44 | CENPU |  |  | 60 | 0.605 | 0.3667 | Yes |
| 45 | CENPK |  |  | 62 | 0.603 | 0.3720 | Yes |
| 46 | TYMS |  |  | 63 | 0.601 | 0.3790 | Yes |
| 47 | CCDC34 |  |  | 65 | 0.595 | 0.3843 | Yes |
| 48 | IGFBP5 |  |  | 67 | 0.590 | 0.3894 | Yes |
| 49 | SMC2 |  |  | 68 | 0.586 | 0.3962 | Yes |
| 50 | CDK1 |  |  | 69 | 0.585 | 0.4031 | Yes |
| 51 | H2AFX |  |  | 71 | 0.576 | 0.4081 | Yes |
| 52 | CKS1B |  |  | 72 | 0.575 | 0.4148 | Yes |
| 53 | UPP1 |  |  | 74 | 0.572 | 0.4197 | Yes |
| 54 | MAD2L1 |  |  | 77 | 0.569 | 0.4230 | Yes |
| 55 | DBF4 |  |  | 79 | 0.567 | 0.4279 | Yes |
| 56 | CTGF |  |  | 81 | 0.564 | 0.4327 | Yes |
| 57 | CKS2 |  |  | 83 | 0.558 | 0.4375 | Yes |
| 58 | UBE2S |  |  | 87 | 0.555 | 0.4389 | Yes |
| 59 | AURKB |  |  | 89 | 0.548 | 0.4436 | Yes |
| 60 | CENPM |  |  | 92 | 0.544 | 0.4465 | Yes |
| 61 | PBK |  |  | 95 | 0.540 | 0.4494 | Yes |
| 62 | DEPDC1 |  |  | 97 | 0.536 | 0.4540 | Yes |
| 63 | SKA3 |  |  | 99 | 0.525 | 0.4584 | Yes |
| 64 | TMEM106C |  |  | 100 | 0.523 | 0.4645 | Yes |
| 65 | RRM1 |  |  | 103 | 0.520 | 0.4671 | Yes |
| 66 | MXD3 |  |  | 104 | 0.519 | 0.4732 | Yes |
| 67 | CMC2 |  |  | 105 | 0.516 | 0.4792 | Yes |
| 68 | UBE2T |  |  | 110 | 0.512 | 0.4784 | Yes |
| 69 | CDCA3 |  |  | 111 | 0.511 | 0.4843 | Yes |
| 70 | DIAPH3 |  |  | 112 | 0.510 | 0.4903 | Yes |
| 71 | TUBB4B |  |  | 114 | 0.507 | 0.4945 | Yes |
| 72 | BIRC5 |  |  | 116 | 0.504 | 0.4986 | Yes |
| 73 | CLSPN |  |  | 117 | 0.503 | 0.5045 | Yes |
| 74 | DTYMK |  |  | 118 | 0.503 | 0.5104 | Yes |
| 75 | ASF1B |  |  | 120 | 0.501 | 0.5145 | Yes |
| 76 | HIST1H2AM |  |  | 121 | 0.497 | 0.5203 | Yes |
| 77 | PSMC3IP |  |  | 122 | 0.495 | 0.5261 | Yes |
| 78 | DUT |  |  | 124 | 0.494 | 0.5301 | Yes |
| 79 | SPDL1 |  |  | 126 | 0.492 | 0.5341 | Yes |
| 80 | TMPO |  |  | 127 | 0.492 | 0.5399 | Yes |
| 81 | SHCBP1 |  |  | 133 | 0.484 | 0.5370 | Yes |
| 82 | TNFRSF12A |  |  | 134 | 0.484 | 0.5426 | Yes |
| 83 | GMNN |  |  | 135 | 0.484 | 0.5483 | Yes |
| 84 | CDCA4 |  |  | 148 | 0.461 | 0.5332 | Yes |
| 85 | ORC6 |  |  | 150 | 0.461 | 0.5369 | Yes |
| 86 | LYPD1 |  |  | 151 | 0.459 | 0.5423 | Yes |
| 87 | KIF22 |  |  | 152 | 0.459 | 0.5476 | Yes |
| 88 | CDC45 |  |  | 154 | 0.456 | 0.5512 | Yes |
| 89 | CEP78 |  |  | 161 | 0.451 | 0.5463 | Yes |
| 90 | TK1 |  |  | 167 | 0.444 | 0.5429 | Yes |
| 91 | ATAD5 |  |  | 170 | 0.442 | 0.5447 | Yes |
| 92 | BRCA2 |  |  | 174 | 0.436 | 0.5447 | Yes |
| 93 | RBBP8 |  |  | 176 | 0.436 | 0.5480 | Yes |
| 94 | MKI67 |  |  | 179 | 0.436 | 0.5497 | Yes |
| 95 | FEN1 |  |  | 180 | 0.435 | 0.5548 | Yes |
| 96 | USP1 |  |  | 185 | 0.430 | 0.5530 | Yes |
| 97 | MIS18BP1 |  |  | 186 | 0.430 | 0.5580 | Yes |
| 98 | CDKN2D |  |  | 190 | 0.427 | 0.5579 | Yes |
| 99 | MND1 |  |  | 193 | 0.424 | 0.5594 | Yes |
| 100 | MIS18A |  |  | 196 | 0.421 | 0.5609 | Yes |
| 101 | DSN1 |  |  | 202 | 0.413 | 0.5572 | Yes |
| 102 | SYNE2 |  |  | 203 | 0.413 | 0.5620 | Yes |
| 103 | TCF19 |  |  | 207 | 0.410 | 0.5617 | Yes |
| 104 | ZWINT |  |  | 212 | 0.407 | 0.5596 | Yes |
| 105 | H2AFZ |  |  | 216 | 0.405 | 0.5593 | Yes |
| 106 | HSPB11 |  |  | 218 | 0.401 | 0.5622 | Yes |
| 107 | VRK1 |  |  | 238 | 0.388 | 0.5344 | No |
| 108 | TRIP13 |  |  | 241 | 0.387 | 0.5356 | No |
| 109 | PCNA |  |  | 242 | 0.386 | 0.5400 | No |
| 110 | HMGN2 |  |  | 243 | 0.386 | 0.5445 | No |
| 111 | RTKN2 |  |  | 248 | 0.379 | 0.5422 | No |
| 112 | RAD23A |  |  | 258 | 0.371 | 0.5312 | No |
| 113 | RFC5 |  |  | 260 | 0.370 | 0.5338 | No |
| 114 | TUBB |  |  | 263 | 0.368 | 0.5347 | No |
| 115 | CENPW |  |  | 267 | 0.366 | 0.5338 | No |
| 116 | TUBG1 |  |  | 268 | 0.365 | 0.5381 | No |
| 117 | SMC1A |  |  | 274 | 0.361 | 0.5338 | No |
| 118 | PSIP1 |  |  | 284 | 0.357 | 0.5226 | No |
| 119 | CALM2 |  |  | 290 | 0.354 | 0.5183 | No |
| 120 | ZWILCH |  |  | 292 | 0.353 | 0.5207 | No |
| 121 | DCBLD2 |  |  | 294 | 0.351 | 0.5231 | No |
| 122 | RFC4 |  |  | 295 | 0.351 | 0.5272 | No |
| 123 | PRKDC |  |  | 306 | 0.344 | 0.5142 | No |
| 124 | CENPN |  |  | 309 | 0.343 | 0.5148 | No |
| 125 | DEK |  |  | 316 | 0.341 | 0.5085 | No |
| 126 | AKAP12 |  |  | 320 | 0.339 | 0.5074 | No |
| 127 | RFC2 |  |  | 331 | 0.333 | 0.4943 | No |
| 128 | HIRIP3 |  |  | 336 | 0.331 | 0.4913 | No |
| 129 | BTG3 |  |  | 337 | 0.331 | 0.4952 | No |
| 130 | EIF5 |  |  | 346 | 0.327 | 0.4854 | No |
| 131 | SPARC |  |  | 350 | 0.325 | 0.4841 | No |
| 132 | HAT1 |  |  | 352 | 0.324 | 0.4861 | No |
| 133 | TMEM237 |  |  | 353 | 0.324 | 0.4899 | No |
| 134 | HES4 |  |  | 360 | 0.322 | 0.4835 | No |
| 135 | LMNB1 |  |  | 363 | 0.321 | 0.4838 | No |
| 136 | WDR34 |  |  | 366 | 0.321 | 0.4841 | No |
| 137 | PARP2 |  |  | 372 | 0.319 | 0.4793 | No |
| 138 | PIN1 |  |  | 379 | 0.314 | 0.4728 | No |
| 139 | DCK |  |  | 383 | 0.313 | 0.4713 | No |
| 140 | ID3 |  |  | 389 | 0.311 | 0.4665 | No |
| 141 | RHEB |  |  | 404 | 0.307 | 0.4462 | No |
| 142 | LRR1 |  |  | 410 | 0.304 | 0.4413 | No |
| 143 | PTTG1 |  |  | 413 | 0.302 | 0.4414 | No |
| 144 | FABP7 |  |  | 414 | 0.302 | 0.4449 | No |
| 145 | C12orf75 |  |  | 433 | 0.296 | 0.4177 | No |
| 146 | TFDP1 |  |  | 444 | 0.294 | 0.4042 | No |
| 147 | TEX30 |  |  | 448 | 0.294 | 0.4025 | No |
| 148 | MZT1 |  |  | 456 | 0.291 | 0.3940 | No |
| 149 | SAE1 |  |  | 463 | 0.289 | 0.3871 | No |
| 150 | TTYH1 |  |  | 469 | 0.287 | 0.3820 | No |
| 151 | RNASEH2A |  |  | 478 | 0.285 | 0.3717 | No |
| 152 | CENPH |  |  | 501 | 0.281 | 0.3375 | No |
| 153 | PSMC3 |  |  | 506 | 0.280 | 0.3340 | No |
| 154 | IFITM3 |  |  | 512 | 0.279 | 0.3288 | No |
| 155 | DNMT1 |  |  | 523 | 0.277 | 0.3150 | No |
| 156 | SAC3D1 |  |  | 529 | 0.276 | 0.3097 | No |
| 157 | ACYP1 |  |  | 551 | 0.273 | 0.2771 | No |
| 158 | CDH2 |  |  | 558 | 0.271 | 0.2701 | No |
| 159 | DONSON |  |  | 559 | 0.271 | 0.2733 | No |
| 160 | GGH |  |  | 564 | 0.270 | 0.2696 | No |
| 161 | CTNNAL1 |  |  | 570 | 0.269 | 0.2642 | No |
| 162 | RPA3 |  |  | 584 | 0.267 | 0.2452 | No |
| 163 | KPNB1 |  |  | 600 | 0.263 | 0.2228 | No |
| 164 | CHCHD3 |  |  | 628 | 0.259 | 0.1799 | No |
| 165 | ARL6IP6 |  |  | 629 | 0.258 | 0.1829 | No |
| 166 | EXOSC8 |  |  | 647 | 0.256 | 0.1570 | No |
| 167 | PXMP2 |  |  | 663 | 0.253 | 0.1344 | No |
| 168 | TUBA1A |  |  | 686 | -0.263 | 0.1001 | No |
| 169 | SOX4 |  |  | 712 | -0.305 | 0.0611 | No |
| 170 | PRDX1 |  |  | 719 | -0.326 | 0.0547 | No |
| 171 | BTG1 |  |  | 741 | -0.423 | 0.0239 | No |
| 172 | MLLT11 |  |  | 744 | -0.430 | 0.0255 | No |
Table: GSEA details [plain text format]

  

Fig 2: FELDMAN\_2020\_S-G2: Random ES distribution      
 Gene set null distribution of ES for **FELDMAN\_2020\_S-G2**
